# Deficiencies in the Intentions, Attitudes, and Knowledge of Future Healthcare Professionals Regarding Breastfeeding

**DOI:** 10.3390/children10071256

**Published:** 2023-07-21

**Authors:** Marija Čatipović, Štefica Mikšić, Rajko Fureš, Zrinka Puharić, Dragica Pavlović

**Affiliations:** 1 Department of Nursing, Bjelovar University of Applied Sciences, Trg E. Kvaternika 4, 43000 Bjelovar, Croatia; zpuharic@vub.hr; 2 Pediatric Office Marija Čatpović, 43000 Bjelovar, Croatia; 3 Faculty of Dental Medicine and Health Osijek, Josip Juraj Strossmayer University of Osijek, 31000 Osijek, Croatia; steficamiksic@gmail.com (Š.M.); rajko.fures@bolnica-zabok.hr (R.F.); dragica.pavlovic@dzobz.hr (D.P.); 4 Department of Gynecology and Obstetrics, Zabok General Hospital and Croatian Veterans Hospital, 49210 Zabok, Croatia

**Keywords:** breastfeeding, education, health studies

## Abstract

The aim of this study was to determine the level of knowledge, positive intentions, and attitudes regarding breastfeeding among university students. A validated questionnaire collected data from 236 students of the Faculty of Dental Medicine and Health Osijek about breastfeeding intentions, attitudes, and knowledge. Descriptive methods were used to present the students’ results in terms of their intentions, attitudes, and knowledge. For each question included in the questionnaire, the maximum possible and maximum achieved numbers of points were calculated, as well as the percentage of points achieved in relation to the maximum possible number. Correlations between the results on individual scales of the questionnaire and the total results of the questionnaire are shown by Spearman’s correlation coefficient. Questionnaire answers that were least in line with professional recommendations were selected and analyzed. We found that the areas that should be given special attention in the education of students are: the intention of breastfeeding for more than a year; the use of breaks for breastfeeding in the workplace; attitudes and knowledge about the quality of breast milk in relation to substitutes; attitudes about breastfeeding in public places and breastfeeding for more than two years; and the feeling of maternity and breastfeeding (compared to bottle feeding). The relationship between the results was considered in terms of intentions, attitudes, and knowledge in relation to the overall results of the questionnaire, and the authors’ thoughts on the reasons behind the poorer results achieved for certain questions were presented.

## 1. Introduction

### 1.1. The Importance of Breastfeeding

Providing support to parents in caring for children is one of the obligations of society cited in the UN Convention on the Rights of the Child [1]. The recommendation of the Council of Europe 19 (2006) on the policy of supporting positive parenting advocates for the development of an integrated, multi-focused, and flexible service in the community [2]. In accordance with the UN Convention on the Rights of the Child, each member state is obliged to inform, educate, and provide support for the use of basic knowledge about children’s health and nutrition. In 2018, Croatia signed the Resolution of the World Health Organization on the nutrition of infants and young children, which recommends exclusive breastfeeding in the first six months of a child’s life and continued breastfeeding with adequate supplementary nutrition from the sixth month of life onwards [3]. Raising breastfeeding rates globally can save hundreds of thousands of lives per year [4]. The benefits of mother’s milk over alternative nutrition are unquestionable. Health benefits for both the child and the mother are emphasized [5,6,7,8,9,10,11,12,13], as are economical [14], emotionally developmental [15], and ecological benefits [16,17]. 

### 1.2. The Factors Influencing the Decision to Breastfeed

The factors influencing the decision to breastfeed are a positive attitude of the mother and father towards breastfeeding; a positive attitude of the local community towards breastfeeding; the provision of help to the breastfeeding mother within the family; previous positive experience with breastfeeding (personal or in a close environment); parents being breastfed as babies; the maturity of the mother; parents having a high level of education; knowledge of the benefits of breastfeeding; socioeconomic status of the family, etc. Health professionals are a crucial factor determining the prevalence of successful breastfeeding in a community. Patients do not expect only theoretical breastfeeding knowledge from healthcare professionals. They expect good, practical implementation of health professionals’ knowledge through an individualized and sensitive approach to breastfeeding mothers and their children [18]. That is why it is important that health professionals, in addition to providing good knowledge, support patients with positive breastfeeding attitudes. Breastfeeding attitudes are formed in adolescence [19]. If we want future healthcare workers to have positive breastfeeding attitudes, it is reasonable to focus educational interventions on that age group. Delayed interventions lead to entrenched negative attitudes that are difficult to change. For this reason, the research is focused on health care students’ intentions, attitudes, and knowledge about breastfeeding.

### 1.3. The Aim of Study

The aim of this study was to determine the gaps between the knowledge, intentions, and attitudes of future healthcare workers regarding breastfeeding. The processing of respondents’ answers will show the areas where students’ knowledge, attitudes, and intentions are the weakest. Therefore, while planning future educational activities, special attention should be paid to these areas. The results of this study will enable better adaptation of the breastfeeding education program to students’ needs, which will prepare them better for their future work on breastfeeding support in medical offices and hospital wards.

## 2. Materials and Methods

### 2.1. Participants

This study is a cross-sectional study. The respondents filled out an online questionnaire about their breastfeeding intentions, attitudes, and knowledge. The results were processed and are presented here. Approval for the research was given by the Ethics Committee of Faculty of Dental Medicine and Health Osijek.

The respondents were future healthcare workers, students of health studies at the Josip Juraj Strossmayer University in Osijek. The research was conducted in the period from 1 February 2020 to 30 April 2020. During the given period, 250 students filled out the online questionnaire. The results of 14 respondents had to be rejected due to incomplete questionnaires. The criteria for inclusion in the study were adulthood and student status in one of the health study programs of Faculty of Dental Medicine and Health Osijek. Students were not supposed to have children (due to the characteristics of the BIAKQ questionnaire), they had to be computer literate in order to access the questionnaire online, and they had to give informed consent to participate in the research. The questionnaire was posted on the website of the Faculty of Dental Medicine and Health Osijek. Students received information about the research and a link to access the questionnaire from the lecturer during their Pediatrics course.

### 2.2. Measurements

The research used a validated questionnaire to assess students’ intentions, attitudes, and knowledge about breastfeeding (Breastfeeding Intentions Attitudes and Knowledge Questionnaire (BIAKQ)). The questionnaire was validated; the procedure and results of the validation are described in detail in the paper entitled Development and Validation of a Questionnaire on Breastfeeding Intentions, Attitudes and Knowledge of a Sample of Croatian Secondary-School Students, which is available online at https://vinkocatipovic.wixsite.com/breastfeeding (accessed on 19 July 2023) [20].

The questionnaire consisted of 10 items on intentions, 32 items on attitudes, and 13 items on knowledge. On the scales of intentions and attitudes, respondents evaluated the statements on a Likert-type scale from 1 to 5 (1—strongly disagree; 2—disagree; 3—neither agree nor disagree; 4—agree; 5—strongly agree). On the knowledge scale, the possible answers were true or false. On the scale of intentions, questions for men were asked in terms of their intentions to support a partner who wishes to breastfeed.

### 2.3. Processing the Results

The interpretation of the results of the BIAKQ questionnaire was carried out in such a way that each answer was evaluated in accordance with the guidelines of the medical profession. On the scale of intentions and attitudes, the best grade, i.e., grade five, indicated an intention or attitude of the respondent that was fully aligned with the recommendations of the profession. An answer that was completely contrary to the guidelines of the profession was scored with the worst grade, i.e., grade one. Reverse evaluation was used for questionnaire items containing negative statements. On the knowledge scale, a correct answer was scored with one point, and an incorrect answer with zero points. Instructions for the examiners were an integral part of the questionnaire. The questionnaire is attached to this paper in the Supplementary Materials.

### 2.4. Data Analysis

The statistical processing began by looking for incompletely filled out questionnaires. This was carried out by visual inspection of the results. After separating the incompletely filled out questionnaires, 236 correctly filled out questionnaires remained. The respondents’ answers to the BIAKQ questionnaire were presented using descriptive statistics methods (frequencies, percentages). The minimum number of points that one respondent could receive on the scale of intentions was 10; on the scale of attitudes, 32; and on the scale of knowledge, 0 (in total per one respondent: 42 points). The maximum number of points on the scale of intentions was 50; on the scale of attitudes, 160; and on the scale of knowledge, 13 (in total per one respondent: 223 points). The maximum possible number of points for all respondents on one item on either the intentions or attitudes scale was 1180 (5 points × 236 respondents), and on the scale of knowledge, 236 (1 point × 236 respondents). The percentage of achieved points shown in the tables represents the quotient of points gained on a particular item and the maximum possible number of points on that item. The total number of points that all respondents could achieve together on the intention scale of the BIAKQ questionnaire was 11,800 (5 points × 236 respondents × 10 items). The total number of points that all respondents could achieve together on the attitude scale of the BIAKQ questionnaire was 37,760 points (5 points × 236 respondents × 32 items). The total number of points that all respondents could achieve together on the knowledge scale of the BIAKQ questionnaire was 3068 points (1 points × 236 respondents × 13 items). The total sum of points achieved through the questionnaire represents the sum of the points achieved on all three scales (52,628 points). Spearman’s correlation coefficient was used to show the correlations between the results of individual scales and the total results of the questionnaire.

## 3. Results

A total of 236 students participated in the research: 97 nursing studies from the city of Nova Gradiška (41.10%), 56 nursing studies from the city of Pregrada (23.73%), and 83 physiotherapy students from the city of Orahovica (35.17%). Most of the respondents were women (205, i.e., 86.44%), while men were in the minority (32, i.e., 13.56%). As for the year of study, there were 69 (29.24%) first-year undergraduate students, 63 (26.69%) second-year undergraduate students, 66 (27.97) third-year undergraduate students, and 38 (16.10%) graduate students.

It can be seen from Table 1 that the respondents obtained the lowest number of points on the questions about the intention to breastfeed after the first (66.02%) and second years of the child’s life (53.22%), and the question on breastfeeding during break time at the workplace (55.51%).

The results shown in Table 2 confirm that the respondents achieved the lowest scores on the following knowledge questions:

– Breastmilk substitutes are not as good as breast milk (57.20%);
– The law should prevent disrupting mothers who breastfeed in public (67.29%);
– It is not good to breastfeed a child for more than two years because it increases the child’s attachment to the mother (66.95%);
– If the mother and the child want it, it is justified to breastfeed a child who is more than two years old (64.75%);
– A mother who feeds her newborn/infant with breast milk substitutes misses a part of maternity enjoyment (65.85%).

Table 3 shows the results of the respondents on the knowledge scale. The respondents had the lowest scores on the following knowledge question: Breastmilk substitutes are just as nutritious and high in quality as breast milk (80.51% achieved the correct score).

The results presented in Table 4 confirm the high correlation of the points achieved on the scale of intentions, the scale of attitudes and the total results of the questionnaire.

## 4. Discussion

Women responded significantly better to the invitation to participate in the research. This is partly due to the lower representation of men in health professions, especially nursing [24], but also in medicine [25]. However, the ratio of male to female participants in the research was significantly lower than the ratio of male students and men to women employed in Croatian healthcare. Therefore, the most likely explanation is that male students had less interest in the subject of breastfeeding and less desire to participate in the research. Most women will give birth in their lifetime; therefore, they need to make plans about breastfeeding their children. The topic of breastfeeding is present in a woman’s mind from the first menstruation [26,27]. 

Intentions are an important predictor of behavior, but behavior is also influenced by other factors. For example, the time distance from the realization of the predicted behavior significantly affects the reliability of behavior prediction based on intention [28]. The reliability of predicting behavior based on intention is also affected by the importance of the decision [29,30]. Most women define their intention to breastfeed before arriving at the maternity hospital [31], which directs research towards younger age groups. The importance of being aware of future healthcare professionals’ intentions about breastfeeding is related to the assumption that their personal intentions may influence the advice they give to patients regarding breastfeeding.

Respondents showed great difficulty in accepting the WHO’s recommendations on breastfeeding after one year, and especially after two years [32]. Breastfeeding requires the mother to adapt to the child’s sleeping and eating rhythms, which is especially demanding during the period of exclusive breastfeeding [33]. Skin-to-skin contact [34] and face-to-face interaction [35], in addition to the characteristics of breast milk, are benefits of breastfeeding when the mother is breastfeeding properly [36]. In contrast to breastfeeding with breast milk substitutes (“using a bottle”), where the mother can be replaced by another person (father of the child, grandmother, aunt, older sister, etc.), when breastfeeding, another person cannot adequately replace the mother. The partner can help the nursing mother in caring for the child (e.g., changing diapers, bathing and carrying the child) and household chores. The mother must, to some extent, give up her own freedom and comfort and submit to the interests and needs of the child. It is possible that such renunciation and responsibility can seem difficult and even frightening to young people, like our respondents. By personally participating in the department’s support activities, future healthcare workers can experience the importance of not only mother’s milk as the healthiest food for newborns and infants, but also breastfeeding itself as the first and most fundamental human relationship. 

In the curriculum, students learn that mother’s milk is dynamic and adaptable to the individual needs of the child on a daily basis [37]. They learn about the immune and nutritional benefits of breast milk [38]. However, in expressing their opinions about the advantages of mother’s milk compared to substitute milk, the students achieved only 57.20% of the possible points. During breastfeeding, a child can explore their mother’s body, take a safe nap, or simply enjoy their mother’s warmth [39]. The neurotransmitters present and hormonal processes that ensue in the body of a breastfeeding mother [40] do not occur with bottle feeding. Future healthcare professionals should accept that bottle feeding cannot achieve the same neurodevelopmental benefits as breastfeeding [41,42]. On the question of the intention to breastfeed “on demand”, the students achieved 72.03 percent of the possible points, even though they learn in class that a newborn knows no delay, and does not predict what will happen “next.” A newborn perceives the deprivation of breastfeeding as a break between them and their mother. It is overwhelmed by an oceanic feeling of horror at the disappearance of protection and security [43]. Breastfeeding is much more than feeding [16,44]. The opinion that it should be legally possible for a mother to breastfeed her child in a public place without disturbance achieved 67.29% of the possible points. Limiting a mother’s right to breastfeed a hungry child in a public place can also be interpreted through traditional gender roles and non-acceptance of the complete identity of a woman [45]. Also, preventing a mother’s from breastfeeding a hungry child in a public place is taking away the hungry child’s right to have its hunger satisfied in the way the child and the mother choose, which health professionals should not support.

Nursing students do not always receive adequate breastfeeding education during their schooling to effectively help mothers. This does not mean only a lack of professional information, but also the ability to acquire practical skills for providing support to nursing mothers and solving the problems and difficulties that arise during breastfeeding [46]. Research in Nigeria has shown that students have sufficient knowledge about breastfeeding; however, only 4 out of every 10 students have a positive attitude towards breastfeeding and 36.6% have intentions to breastfeed in the future [47]. The Jordanian study singled out the deficiencies in the students’ knowledge about the management of breast problems such as mastitis, cracked nipples, milk oversupply, appropriate conditions for weaning, and insecurity of midwifery students in their abilities to assist women. The authors emphasized the importance of focusing on the development of supportive and positive attitudes [48]. The study conducted in Saudi Arabia identified specific gaps in knowledge and attitudes that pertain mainly to breastfeeding in public, i.e., perceptions that breastfeeding is painful; that formula feeding gives more freedom to the mother; and that dietary restrictions by the mother are needed during breastfeeding [49]. 

The findings of the authors of this study coincide with the works of Yang [46] and Lesch [47]. It is important to emphasize that the Croatian school curriculum includes the knowledge necessary for students to correctly answer the questions asked in the questionnaire. Looking carefully at the results of this research, we found that the students had mastered most of the information and achieved an excellent result on the knowledge scale (they achieved as much as 93.02% of the maximum possible points). This means that, during their education, a good result was achieved in terms of acquiring knowledge. However, the respondents scored much lower on the scale of attitudes (78.49%), and even lower on the scale of intentions (72.33%). There is a clear discrepancy between the results on the scale of knowledge and the results on the scales of attitudes and intentions. Therefore, the authors of the study searched for the cause of the poor results achieved by the students on certain parts of the questionnaire in terms of the personal intentions and attitudes of the respondents (and not in the lack of theoretical knowledge of the students). This was also confirmed by the high correlation of the total results of the questionnaire with the results on the intention and attitude scales, and the low correlation with the results on the knowledge scale. An attitude is an acquired, relatively permanent, and stable structure of positive or negative emotions, evaluation, and behavior towards an object [50]. We can define attitude as a function of expressing self-image and, thus, as a function of ego defense [51]. According to this interpretation, the relationship between attitude and behavior is reciprocal, meaning that already adopted behavior shapes one’s attitude in such a way that it justifies the behavior, in front of both oneself and other people. Ingrained attitudes that have already been expressed through behavior are difficult to change, because practiced behavior (created on the basis of these attitudes) makes the process of changing one’s attitude difficult. It is possible (but not proven by this study) that healthcare professionals who have given up breastfeeding their own children will have greater difficulties in providing support to parents and infants regarding breastfeeding compared to a healthcare professional with a positive experience of breastfeeding their own child. 

If we were to accept the presented assumption about the influence of personal intentions and attitudes on the results of the respondents, it would mean that the education of future health workers should be organized in such a way that they become familiar with their own intentions and attitudes about breastfeeding. Lectures aimed at conveying knowledge from experts are necessary, but not sufficient. Personal emotional experience is required. A new emotional experience leads to emotional cognition, i.e., so-called experienced knowledge, which, when combined with existing cognitive knowledge, can lead to reconsideration of attitudes formed on old comprehensions and experience. In the case of inconsistency with cognitive knowledge, attitudes formed according to old understandings and experiences are re-examined. This creates a basis for potential changes in behavior. Future healthcare professionals can gain new emotional experience by means of direct (supervised) work with pregnant women and mothers in maternity hospitals, pediatric surgeries in primary health care, visiting nurse services, breastfeeding support groups, and public breastfeeding support activities in cooperation with civil society associations [52].

The advantage of this research is that it successfully singled out the topics regarding which future healthcare workers (assessing their attitudes, intentions and knowledge) gave the worst answers. The study resulted in the hypothesis that the possible cause of disappointing results in certain parts of the questionnaire is resistance based on the personal attitudes and intentions of the students. The limitation of this study is that it only points out the problem, but does not offer a proven solution to the problem that can be used in practice. In their defense, the authors state that it is difficult to prove the cause and to offer a verified and effective solution to the problem with cross-sectional research. The results of the study should be understood as a step in the right direction in terms of further research and practical work. The authors are working on a study that will more clearly show the influence of personal attitudes and experience on the behavior of healthcare professionals in support of breastfeeding. The plan for the study is to separate two groups of healthcare workers (with positive and negative personal experiences of breastfeeding) and to analyze the differences in the effectiveness of breastfeeding support. 

## 5. Conclusions

This study singled out the areas in which respondents achieved poor results on the questionnaire regarding intentions, attitudes, and knowledge regarding breastfeeding. The results of the research point to the assumption that the inconsistency of one’s own intentions and attitudes about breastfeeding in regard to knowledge (of the benefits of breast milk and breastfeeding) can cause serious difficulties for future health workers in terms of their intentions to apply that knowledge. Possible consequences of this discrepancy include difficulties in working with mothers who are trying to breastfeed their children. Women approach healthcare professionals with great confidence, seeking advice and support [18]. Vague, ambiguous, or uncertain answers and comments from healthcare professionals about breastfeeding are a serious obstacle to successful breastfeeding [53]. The authors of this study do not believe that only healthcare professionals with positive personal experiences of breastfeeding should promote and support breastfeeding. The authors believe that educational work with future healthcare workers should be enriched with activities that will help them to reconsider their personal attitudes and intentions about breastfeeding. This can be achieved through new emotional experiences gained through supervised work with parents who breastfeed their children.

## Figures and Tables

**Table 1:** Responses to the intention scale of the BIAKQ questionnaire (N = 236).

		Respondents						
Items of the Intention Scale	*n* (%)	Strongly Agree	Agree	Neither Agree nor Disagree	Disagree	Strongly Disagree	Total	Points Achieved *
After the delivery, I would not try to establish breastfeeding. I would bottle-feed our child (with breast milk substitutes).								
	*n* (%)	2 (0.85)	2 (0.85)	13 (5.51)	**31 (13.14)**	**188 (79.66)**	236 (100)	1109 (93.98)
I would stop breastfeeding my child as soon as I started to work, even if the child would still want to be breastfed.								
	*n* (%)	10 (4.24)	14 (5.93)	40 (16.95)	**57 (24.15)**	**115 (48.73)**	236 (100)	961 (81.44)
I would not breastfeed in public (for example at a restaurant or in the park) even if the child wanted it.								
	*n* (%)	13 (5.51)	24 (10.17)	52 (22.03)	**47 (19.92)**	**100 (42.37)**	236 (100)	905 (76.69)
After returning to work, I would instantly start to bottle-feed the baby (with breast milk substitutes).								
	*n* (%)	10 (4.24)	16 (6.78)	63 (26.69)	**71 (30.08)**	**76 (32.20)**	236 (100)	895 (75.85)
I would continue to breastfeed after the child turns one if the child would want it.								
	*n* (%)	**67 (28.39)**	**30 (12.71)**	70 (29.66)	45 (19.07)	24 (10.17)	236 (100)	779 (66.02)
Regardless of where I am (home, park, facility) if our child demands breastfeeding, I would breastfeed.								
	*n* (%)	**84 (35.59)**	**49 (20.96)**	66 (27.97)	26 (11.02)	11 (4.66)	236 (100)	877 (74.32)
Returning to work would not make me stop breastfeeding.								
	*n* (%)	**80 (33.9)**	**62 (26.27)**	55 (23.31)	24 (10.17)	15 (6.36)	236 (100)	876 (74.24)
I would not breastfeed our child after he/she turns two, even if the child would want to continue.								
	*n* (%)	63 (26.69)	49 (20.76)	68 (28.81)	**17 (7.20)**	**39 (16.53)**	236 (100)	628 (53.22)
If my partner would be helping me by bringing the child to my workplace, I would breastfeed during break time.								
	*n* (%)	**30 (12.71)**	**38 (16.10)**	66 (27.97)	53 (22.46)	49 (20.76)	236 (100)	655 (55.51)
I intend to breastfeed “on demand”, and not on fixed terms (e.g., every 3–4 h).								
	*n* (%)	**73 (30.93)**	**56 (23.73)**	66 (27.97)	22 (9.32)	19 (8.05)	236 (100)	850 (72.03)

* The total number of points that all respondents achieved together on the intention scale BIAKQ questionnaire was 8535 (72.33% of the maximum possible number of points). Answers that are in line with professional recommendations are highlighted in bold.

**Table 2:** Responses to the attitude scale of the BIAKQ questionnaire (N = 236).

		Respondents						
Items of the Attitude Scale	*n*	Strongly Agree	Agree	Neither Agree, nor Disagree	Disagree	Strongly Disagree	Total	Points Achieved *
It is more pleasant to see a mother feeding the baby with a bottle than breastfeeding.								
	*n* (%)	5 (2.12)	17 (7.20)	50 (21.19)	**57 (24.15)**	**107 (45.34)**	236 (100)	952 (80.68)
Breastfeeding has a long-term negative effect on the mother’s working abilities and career.								
	*n* (%)	2 (0.85)	6 (2.54)	28 (11.86)	**53 (22.46)**	**147 (62.29)**	236 (100)	1045 (88.56)
I think a partner should help a working nursing mother by bringing the child to her during work breaks.								
	*n* (%)	**59 (25.00)**	**53 (22.46)**	74 (31.36)	31 (13.14)	19 (8.05)	236 (100)	810 (68.64)
It is not profitable to invest in breastfeeding.								
	*n* (%)	0 (0,00)	3 (1.27)	19 (8.05)	**46 (19.49)**	**(71.19)**	236 (100)	1087 (92.12)
Partners attending breastfeeding support groups can learn how to help mothers in starting and maintaining breastfeeding.								
	*n* (%)	**109 (46.19)**	**86 (36.44)**	27 (11.44)	10 (4.24)	4 (1.69)	236 (100)	994 (84.24)
Breastfeeding in public should be prohibited.								
	*n* (%)	5 (2.12)	7 (2.97)	33 (13.98)	**36 (15.25)**	**155 (65.68)**	236 (100)	1037 (87.88)
It is wrong to breastfeed a child who is more than one year old.								
	*n* (%)	5 (2.10)	18 (7.60)	76 (32.20)	**40 (16.90)**	**97 (41.10)**	236 (100)	914 (77.46)
A mother who feeds her newborn/infant with breast milk substitutes misses a part of maternity enjoyment.								
	*n* (%)	**51 (21.61)**	**46 (19.49)**	86 (36.44)	27 (11.44)	26 (11.02)	236 (100)	777 (65.85)
The child’s father should necessarily use part of the parental leave to help the mother with breastfeeding and childcare.								
	*n* (%)	**68 (28.81)**	**70 (29.66)**	74 (31.36)	15 (6.36)	9 (3.81)	236 (100)	881 (74.66)
Women should not breastfeed in public.								
	*n* (%)	10 (4.24)	12 (5.08)	46 (19.49)	**46 (19.49)**	**122 (51.69)**	236 (100)	966 (81.86)
An employer should provide a space where the employed mother can breastfeed her child or use a breast pump without interruption, regardless of whether he or she is obliged to do so by law.								
	*n* (%)	**70 (29.66)**	**60 (25.42)**	69 (29.24)	25 (10.59)	12 (5.08)	236 (100)	859 (72.80)
Breastfeeding in public is natural.								
	*n* (%)	**83 (35.17)**	**64 (27.12)**	59 (25.00)	17 (7.20)	13 (5.51)	236 (100)	895 (75.85)
If the mother and the child want it, it is justified to breastfeed a child who is more than two years old.								
	*n* (%)	**50 (21.19)**	**50 (21.19)**	72 (30.51)	34 (14.41)	30 (12.71)	236 (100)	764 (64.75)
Being informed about breastfeeding can significantly help the father in aiding the breastfeeding mother.								
	*n* (%)	**133 (56.36)**	**77 (32.63)**	17 (7.20)	6 (2.54)	3 (1.27)	236 (100)	1039 (88.05)
On the day of delivery, the mother should not breastfeed the child because she needs to rest.								
	*n* (%)	4 (1.69)	6 (2.54)	54 (22.88)	**40 (16.95)**	**132 (55.93)**	236 (100)	998 (84.58)
It is okay for a woman to breastfeed at the workplace during a breastfeeding break.								
	*n* (%)	**64 (27.12)**	**60 (25.42)**	75 (31.78)	22 (9.32)	15 (6.36)	236 (100)	844 (71.53)
The father does not play a key role in the child’s life while the child is breastfed.								
	*n* (%)	2 (0.85)	0	8 (3.39)	**29 (12.29)**	**197 (83.47)**	236 (100)	1127 (95.51)
It is not good to breastfeed a child for more than two years because it increases the child’s attachment to the mother.								
	*n* (%)	21 (8.90)	22 (9.32)	102 (43.22)	**36 (15.25)**	**55 (23.31)**	236 (100)	790 (66.95)
People who have had the opportunity to see a woman breastfeeding in public are more willing to breastfeed in public themselves or support public breastfeeding.								
	*n* (%)	**59 (25.00)**	**68 (28.81)**	82 (34.75)	17 (7.20)	10 (4.24)	236 (100)	857 (72.63)
One of the father’s roles in the first year of the child’s life is to be supportive and helpful to the mother.								
	*n* (%)	**176 (74.58)**	**39 (16.53)**	11 (4.66)	4 (1.69)	6 (2.54)	236 (100)	1083 (91.78)
A mother should breastfeed the child for the first time on the second day after giving birth.								
	*n* (%)	7 (2.97)	16 (6.78)	65 (27.54)	**43 (18.22)**	**105 (44.49)**	236 (100)	931 (78.90)
Children who are fed breast milk are healthier than children who are fed breast milk substitute.								
	*n* (%)	**92 (38.98)**	**68 (28.81)**	43 (18.22)	19 (8.05)	14 (5.93)	236 (100)	913 (77.37)
One of the important tasks of the child’s father after the birth of the child is to monitor the condition of the child’s mother and ensure that she eats enough and rests.								
	*n* (%)	**136 (57.63)**	**65 (27.54)**	26 (11.02)	4 (1.69)	5 (2.12)	236 (100)	1031 (87.37)
Breastfeeding in public spreads and promotes the culture of breastfeeding as the best food for a child.								
	*n* (%)	**91 (38.56)**	**75 (31.78)**	48 (20.34)	16 (6.78)	6 (2.54)	236 (100)	937 (79.41)
A man feels neglected next to his breastfeeding wife.								
	*n* (%)	3 (1.27)	3 (1.27)	39 (16.53)	**58 (24.58)**	**133 (56.36)**	236 (100)	1023 (86.69)
Breast milk substitutes are not as good as breast milk.								
	*n* (%)	**44 (18.64)**	**39 (16.53)**	87 (36.86)	38 (16.10)	28 (11.86)	236 (100)	675 (57.20)
The law should prevent disrupting the mother who breastfeeds in public.								
	*n* (%)	**44 (18.64)**	**55 (23.31)**	95 (40.25)	27 (11.44)	15 (6.36)	236 (100)	794 (67.29)
Only the child’s mother needs to learn about breastfeeding and the impact of breastfeeding on the child’s development because the child is a woman’s responsibility.								
	*n* (%)	5 (2.12)	9 (3.81)	32 (13.56)	**56 (23.73)**	**134 (56.78)**	236 (100)	1013 (85.85)
Breastfeeding in public increases tolerance and understanding of breastfeeding.								
	*n* (%)	**76 (32.20)**	**82 (34.75)**	52 (22.03)	16 (6.78)	10 (4.24)	236 (100)	906 (76.78)
A mother must not breastfeed the child on the day of delivery.								
	*n* (%)	2 (0.85)	5 (2.12)	68 (28.81)	**36 (15.25)**	**125 (52.97)**	236 (100)	985 (83.47)
Breastfeeding in public is a part of breastfeeding promotion.								
	*n* (%)	**73 (30.93)**	**67 (28.39)**	65 (27.54)	19 (8.05)	12 (5.08)	236 (100)	878 (74.41)
A mother should breastfeed her child for the first time within an hour of the child’s birth.								
	*n* (%)	**64 (27.12)**	**42 (17.80)**	98 (41.53)	18 (7.63)	14 (5.93)	236 (100)	832 (70.51)

* The total number of points that all respondents achieved together on the attitude scale of the BIAKQ questionnaire was 29,637 (78.49% of the maximum possible number of points). Answers that are in line with professional recommendations are highlighted in bold.

**Table 3:** Responses to the knowledge scale of the BIAKQ questionnaire (N = 236).

Items of the Knowledge Scale		FALSE	TRUE	Total
Children fed with breast milk substitutes are healthier than breastfed children.	*n* (%)	**214 (90.68)**	22 (9.32)	236
Breast milk provides the child with the best protection against infections.	*n* (%)	8 (3.39)	**228 (96.61)**	236
Breastfeeding has a positive effect on health throughout life, not just during childhood.	*n* (%)	21 (8.90)	**215 (91.10)**	236
Suckling breast milk is not only an instinctive action, but an emotional and developmental need of the child.	*n* (%)	3 (1.27)	**233 (98.73)**	236
Breastfeeding increases disease risk for the mother.	*n* (%)	**232 (98.31)**	4 (1.69)	236
Breastfeeding has been shown to be beneficial for the development of emotional attachment between the mother and her child.	*n* (%)	2 (0.85)	**234 (99.15)**	236
The iron in breast milk has a low capacity for reabsorption [21].	*n* (%)	**216 (91.53)**	20 (8.47)	236
If the child is fed with breast milk substitutes in the maternity hospital, it is not possible to establish successful breastfeeding upon returning home.	*n* (%)	**219 (92.80)**	17 (7.20)	236
Breastfeeding reduces disease risk for the child.	*n* (%)	12 (5.08)	**224 (94.92)**	236
Breast milk substitutes are just as nutritious and high in quality as breast milk.	*n* (%)	**190 (80.51)**	46 (19.49)	236
Breast milk has no medicinal properties.	*n* (%)	**220 (93.22)**	16 (6.78)	236
Breastfeeding protects the child from infectious diseases and presumably from allergies [22].	*n* (%)	19 (8.05)	**217 (91.95)**	236
Breastfeeding promotes healthy cognitive development [23].	*n* (%)	24 (10.17)	**212 (89.83)**	236

The TOTAL number of points that all respondents achieved together on the knowledge scale of the BIAKQ questionnaire was 2854 (93.02% of the maximum possible number of points). Answers that are in line with professional recommendations are highlighted in bold.

**Table 4:** Correlations of respondents’ points on the scales of intention, attitudes, and knowledge, as well as the total results of the questionnaire (N = 236).

		Intentions	Attitudes	Knowledge
Attitudes	Spearman’s rho	0.73		
	*p*	<0.00		
Knowledge	Spearman’s rho	0.28	0.26	
	*p*	<0.00	<0.00	
Total	Spearman’s rho	0.88	0.96	0.31
	*p*	<0.00	<0.00	<0.00

## Data Availability

The data presented in this study are available upon request from the corresponding author. The authors do not have the consent of the participants for public distribution of the database.

## Supplementary Materials

The following supporting information can be downloaded at: https://www.mdpi.com/article/10.3390/children10071256/s1, Table S1: The Breastfeeding Intention Attitudes and Knowledge Questionnaire.
