# Supplementary material for: Deficiencies in the Intentions, Attitudes, and Knowledge of Future Healthcare Professionals Regarding Breastfeeding
Source: Children (Basel). 2023 Jul 21;10(7):1256. doi: 10.3390/children10071256 (PMC10378666; doi:10.3390/children10071256)
Supplement: Supplementary file 1 [file children-10-01256-s001.zip › children-2476274-supplementary.pdf]

Table S1. The Breastfeeding Intention Attitudes and Knowledge Questionnaire.

Question number:	BRESTFEEDING INTENTION SACLE (WOMEN)	Strongly disagree	Disagree	Neither disagree nor agree	Agree	Strongly agre
1.	After the delivery, I wouldn't try to establish breastfeeding. I would feed our child with the milk substitute.	5	4	3	2	1
2.	I would stop breastfeeding my child as soon as I started to work, even if a child still desires to breastfeed.	5	4	3	2	1
3.	I wouldn't breastfeed in public, for example in a restaurant or in a park, even if a child desired so.	5	4	3	2	1
4.	After returning to work, I would instantly started to feed our child with the milk substitute.	5	4	3	2	1
5.	I would continue with breastfeeding after a child turns one if a child so desires.	1	2	3	4	5
6.	Regardless of where I am (home, park, facility) if our child demands breastfeeding, I would breastfeed.	1	2	3	4	5
7.	Returning to work would not make me stop breastfeeding.	1	2	3	4	5
8.	I would not breastfeed our child after he/she turns two, even if a child so desires.	5	4	3	2	1
9.	If my partner would have helped me and brought child to my workplace, I would breastfeed during breastfeeding pause.	1	2	3	4	5
10.	I intend to breastfeed "on a demand", not fixed-term (eg every 3-4 hours).	1	2	3	4	5

Question number:	BRESTFEEDING INTENTION SACLE (PARTNER)	Strongly disagree	Disagree	Neither disagree nor agree	Agree	Strongly agre
1.	I wouldn't support the mother of our child to try to breastfeed after birth, but to immediately begin feeding the baby with a milk substitute ("on the bottle").	5	4	3	2	1
2.	I would support the mother of our child to stop breastfeeding our child as soon as she started to work, even if a child still desires to breastfeed.	5	4	3	2	1
3.	I wouldn't support the mother of our child to breastfeed in the public, for example in a restaurant or in a park, even if the child was hungry and looking for help.	5	4	3	2	1
4.	I will try to encourage the mother of our child to instantly start to bottle-feed the baby with a milk substitute after returning to work.	5	4	3	2	1
5.	I will support my baby's mother to breastfeed after one year, if both of them wanted to.	1	2	3	4	5
6.	Regardless of where the mother of our child is (house, park, institution etc.), if our baby is hungry and looking for help, I will support baby's mother to breastfeed.	1	2	3	4	5
7.	I will support baby's mother to continue breastfeeding after she returns to work.	1	2	3	4	5
8.	I will not support our baby's mother to breastfeed after the baby is two years old, even if our baby still looked for a breast.	5	4	3	2	1
9.	I intend to help baby's mother to breastfeed after returning to work, for example, by driving a child to her job during breastfeeding breaks.	1	2	3	4	5
10.	I will support the baby's mother to breastfeed "on a demand", not fixed-term (eg every 3-4 hours).	1	2	3	4	5

Question number:	BRESTFEEDING ATTITUDES SCALE	Strongly disagree	Disagree	Neither disagree nor agree	Agree	Strongly agre
1.	In my opinion it is more pleasant to see mothers who feed their child with bottle / milk substitute than mothers who breastfeed.	5	4	3	2	1
2.	I think that partner should help a breastfeeding mother who works with bringing a baby to her during the breastfeeding pause.	1	2	3	4	5
3.	Breastfeeding long-term negatively affects the mother's working abilities and career.	5	4	3	2	1
4.	It is not profitable to invest in breastfeeding (neither personally or socially).	5	4	3	2	1
5.	Partners attending breastfeeding support groups can learn how to help mothers in starting and maintaining breastfeeding.	1	2	3	4	5
6.	Breastfeeding in public should be prohibited.	5	4	3	2	1
7.	It is wrong to breastfeed a child older than one year.	5	4	3	2	1
8.	A mother who feeds her newborn/infant with breast milk substitutes misses a part of maternity enjoyment..	1	2	3	4	5
9.	It is in the interest of the child and the father that each father spends as much time as possible with the child, and if he is employed, he uses the right to parental leave.	1	2	3	4	5
10.	Women should not breastfeed in public.	5	4	3	2	1
11.	The employer should provide a space where the employed mothers can breastfeed their child or use a breast pump without interruption, regardless of whether or not he or she is obliged to do so by law.	1	2	3	4	5
12.	Breastfeeding in public is natural.	1	2	3	4	5
13.	It is justified to breastfeed the child after the child is 2 years old, if the mother and the child want it.	1	2	3	4	5

14.	The knowledge of breastfeeding could be of a great help to a partner in helping mother who breastfeeds.	1	2	3	4	5
15.	On the day of delivery, the mother should not breastfeed because she needs to rest.	5	4	3	2	1
16.	I agree that it is right for a woman to breastfeed at her workplace during a breastfeeding break.	1	2	3	4	5
17.	Father does not play an important role in the child's life while child breastfeeds.	5	4	3	2	1
18.	It is not good to breastfeed a baby for more than two years because it increases the attachment of the baby to the mother.	5	4	3	2	1
19.	People who have had the opportunity to see a woman who breastfeeds in the public, are more willing to breastfeed in public themselves.	1	2	3	4	5
20.	One of the roles of a father in child´s first year of life is to provide support and all the necessary help to the mother.	1	2	3	4	5
21.	The mother should give a first breastfeed to the baby the day after delivery.	1	2	3	4	5
22.	Children fed with mother's milk are healthier than babies fed with the milk formula.	1	2	3	4	5
23.	The duty of a partner is to monitor the condition of child`s mother and make sure that she is eating and resting enough.	1	2	3	4	5
24.	Breastfeeding in public expands and promotes breastfeeding nutrition culture as the best food for a child.	1	2	3	4	5
25.	The partner feels neglected while the child`s mother is breastfeeding.	5	4	3	2	1
26.	Breast milk substitutes are not as good as breast milk.	1	2	3	4	5
27.	The law should prevent disruption of the mother who breastfeeds in public.	1	2	3	4	5
28.	Only child`s mother needs to learn about breastfeeding and the impact of breastfeeding on child development since a child is only her´s responsibility.	5	4	3	2	1

29.	Breastfeeding in public increases the tolerance and understanding of breastfeeding.	1	2	3	4	5
30.	The mother should not breastfeed the child at the day of a birth.	5	4	3	2	1
31.	Breastfeeding in public is a part of breastfeeding promotion.	1	2	3	4	5
32.	The mother should first breastfeed her baby within an hour of giving a birth.	1	2	3	4	5

Question number:	KNOWLEDGE ABOUT BREASTFEEDING	TRUE	FALSE
1.	Children fed with a substitute formula are healthier than children fed with mother's milk.	0	1
2.	Mother's milk provides the child with the best protection against infections.	1	0
3.	Breastfeeding has a life- lasting positive impact on health, not just in childhood.	1	0
4.	Suckling mother's milk is not only an instinctive action but also an emotional and development need of the child.	1	0
5.	Breastfeeding increases the risk of disease for mother.	0	1
6.	Breastfeeding has proven useful for the development of emotional attachment of mother and child.	1	0
7.	The iron in breast milk has a low capacity for reabsorption.	1	0
8.	If a child is fed with milk formula in nursery, it is not possible to successfully establish breastfeeding when returning back home.	0	1
9.	Breastfeeding reduces the risk of childhood illness.	1	0
10.	Breast milk substitutes are just as nutritious and high in quality as breast milk.	0	1
11.	Mother's milk has no healing properties.	0	1
12.	Breastfeeding protects the child from infectious diseases and presumably from allergies.	1	0
13.	Breastfeeding promotes the cognitive development of children.	1	0

Čatipović, M.; Marković, M.; Grgurić, J. Development and Validation of a Questionnaire on Breastfeeding Intentions, Attitudes and Knowledge of a Sample of Croatian Secondary-School Students. *Children* **2018**, *5*, 56. https://doi.org/10.3390/children5050056.
